# Crosstalk between β-Catenin and CCL2 Drives Migration of Monocytes towards Glioblastoma Cells

**DOI:** 10.3390/ijms23094562

**Published:** 2022-04-20

**Authors:** Philippe Aretz, Donata Maciaczyk, Suad Yusuf, Rüdiger V. Sorg, Daniel Hänggi, Hongjia Liu, Hongde Liu, Tikam Chand Dakal, Amit Sharma, Ramakrishna Bethanabatla, Silke Neumann, Jarek Maciaczyk

**Affiliations:** 1 Department of Neurosurgery, University Hospital Düsseldorf, 40225 Dusseldorf, Germany; philippe.aretz@med.uni-duesseldorf.de (P.A.); suad.yusuf@uni-duesseldorf.de (S.Y.); daniel.haenggi@med.uni-duesseldorf.de (D.H.); 2 Department of Pathology, University of Otago, Dunedin 9016, New Zealand; donata.maciaczyk@otago.ac.nz (D.M.); silke.neumann@otago.ac.nz (S.N.); 3 Institute for Transplantation Diagnostics and Cell Therapeutics, University Hospital Düsseldorf, 40225 Dusseldorf, Germany; ruediger.sorg@med.uni-duesseldorf.de; 4 State Key Laboratory of Bioelectronics, School of Biological Science & Medical Engineering, Southeast University, Nanjing 210096, China; liuhongjia@seu.edu.cn (H.L.); 101100344@seu.edu.cn (H.L.); 5 Genome and Computational Biology Lab, Department of Biotechnology, Mohanlal Sukhadia University, Udaipur 313001, India; tc.dakal@mlsu.ac.in; 6 Department of Stereotactic and Functional Neurosurgery, University Hospital Bonn, 53127 Bonn, Germany; amit.sharma@ukbonn.de; 7 Northumbria Healthcare NHS Foundation Trust, Newcastle upon Tyne NE27 0QJ, UK; ramak@doctors.net.uk; 8 Department of Surgical Sciences, University of Otago, Dunedin 9016, New Zealand

**Keywords:** glioblastoma, GSCs, β-catenin, Wnt, CCL2, monocytes, immune evasion, MSAB

## Abstract

Isocitrate dehydrogenase (IDH)-wildtype glioblastoma (GBM) is a fast growing and highly heterogeneous tumor, often characterized by the presence of glioblastoma stem cells (GSCs). The plasticity of GSCs results in therapy resistance and impairs anti-tumor immune response by influencing immune cells in the tumor microenvironment (TME). Previously, β-catenin was associated with stemness in GBM as well as with immune escape mechanisms. Here, we investigated the effect of β-catenin on attracting monocytes towards GBM cells. In addition, we evaluated whether CCL2 is involved in β-catenin crosstalk between monocytes and tumor cells. Our analysis revealed that shRNA targeting β-catenin in GBMs reduces monocytes attraction and impacts CCL2 secretion. The addition of recombinant CCL2 restores peripheral blood mononuclear cells (PBMC) migration towards medium (TCM) conditioned by shβ-catenin GBM cells. CCL2 knockdown in GBM cells shows similar effects and reduces monocyte migration to a similar extent as β-catenin knockdown. When investigating the effect of CCL2 on β-catenin activity, we found that CCL2 modulates components of the Wnt/β-catenin pathway and alters the clonogenicity of GBM cells. In addition, the pharmacological β-catenin inhibitor MSAB reduces active β-catenin, downregulates the expression of associated genes and alters CCL2 secretion. Taken together, we showed that β-catenin plays an important role in attracting monocytes towards GBM cells in vitro. We hypothesize that the interactions between β-catenin and CCL2 contribute to maintenance of GSCs via modulating immune cell interaction and promoting GBM growth and recurrence.

## 1. Introduction

Despite multimodal treatment including supramarginal resection, radiotherapy and chemotherapy isocitrate dehydrogenase (IDH)-wildtype glioblastoma (GBM), the most common malignant primary brain tumor has a median survival of less than two years [1,2]. Therapy resistance and recurrence tendency in GBM have been attributed to the presence of GSCs [3,4,5,6,7,8,9,10], which promote cancer initiation and progression [11,12,13,14,15,16]. GSCs have been characterized by special metabolic [17] and immunologic behavior [18]. An important intracellular pathway inducing stem cell properties in GBM is the canonical Wnt/β-catenin signaling [19,20,21,22,23,24,25,26,27,28], a highly conserved pathway, which directs cell development, migration and polarity during embryonic development and in carcinogenesis. In GBM, Wnt/β-catenin drives glioblastoma cell survival, migration and maintenance of GSCs [19,20,21,22,23,24,25,26,27,28]. Furthermore, recent evidence indicates that β-catenin—the pathway’s key protein—leads to the exclusion of immune cells from the tumor environment of different cancer types, thus preventing anti-tumor immunity [29,30,31,32,33]. 

Monocyte-derived tumor-associated macrophages (TAMs) and myeloid-derived suppressor cells (MDSC) are commonly found in the GBM tumor mass, with TAMs being the dominant GBM infiltrating immune cell population [34,35,36,37,38,39]. Functionally, TAMs promote tumor growth and metastasis by impairing the anti-tumor immune response [40,41,42,43,44,45,46], among others, by producing chemokines such as C–C motif chemokine ligand 2 (CCL2) [47,48,49]. CCL2 possesses both tumor-inhibitory and tumor-promoting effects, depending on the interaction between cancer and host cells [47,50,51,52,53]. Originally known as a monocyte chemoattractant and pro-inflammatory protein, it has also been shown to drive angiogenesis and metastasis in the TME of different cancer types including GBM [47,54,55,56,57,58,59,60,61].

A positive correlation of GSCs and TAMs has been observed in GBM, suggesting an important role of GSCs in TAM-recruitment [62,63]. A recent study demonstrated that a β-catenin-CCL2 feedback loop mediates crosstalk between cancer cells and macrophages in breast cancer stem cells [64]. Given that the Wnt/β-catenin pathway is active in GSCs [19,20,21,22,23,24,25,26,27,28], we investigated the effect of β-catenin signaling on monocyte migration and potential involvement of CCL2 in β-catenin-dependent cross-talk between monocytes and GBM cells. Because of the numerous molecular and genomic differences between adult and pediatric GBM, we used two adult and one pediatric cell lines for comparison. Furthermore, we performed pharmacological targeting of β-catenin with the small molecule inhibitor (Methyl 3-{(4methylphenyl)sulfonylamino}benzoate, MSAB) [65] to evaluate Wnt/β-catenin inhibition and apoptosis-inducing ability in this context. 

## 2. Material and Methods

### 2.1. Cell Culture and MSAB Treatment

We used three GBM cell lines: GBM1 (adult male, classical subtype, MGMT methylated, IDH wild type) was generously provided by A. Vescovi (Milan, Italy) JHH520 (adult female, mesenchymal subtype, MGMT methylated, IDH wild type) was provided by G. Riggins (Johns Hopkins Hospital Baltimore, Baltimore, MD, USA) and SF188 (8-year-old male, MGMT unmethylated, IDH wild type) was provided by C. Eberhart (Johns Hopkins Hospital Baltimore, Baltimore, MD, USA). HEK293T were purchased from American Tissue Culture Collection (Manassas, VA, USA). All GBM cell lines were cultivated in neurosphere medium containing 70% DMEM w/o pyruvate and 30% Ham’s F12 nutrient mix (both Gibco BRL, Eggenstein, Germany), supplemented with 2% serum free B27 (Gibco BRL), 20 ng/mL bovine fibroblast growth factor, 20 ng/mL human epidermal growth factor (both Peprotech, Rocky Hill, NJ, USA), 5 µg/mL heparin (Sigma-Aldrich, St. Louis, MO, USA) and 1% Anti-Anti Penicillin-Streptomycin Fungizone^®^ mixture (Gibco). HEK293T cells were cultivated in DMEM with pyruvate (Gibco) supplemented with 10% Fetal Calf Serum (FCS; Biochrome, MD, USA) and 1% Anti-Anti Penicillin Streptomycin Fungizone^®^ mixture (Gibco). Cells were cultured under standard conditions (37 °C, 5% CO_2_), and routinely tested for mycoplasma contamination using the PCR-based Mycoplasma Test Kit I/C from Promokine (Heidelberg, Germany) MSAB (Sigma-Aldrich) was diluted in DMSO (Sigma-Aldrich) and stored at −20 °C. For apoptosis assay, immunoblotting and ELISA, cells were cultured for 24 h under general cell culture conditions in the presence of various concentrations of MSAB diluted in neurosphere medium. 

### 2.2. Generation of Lentiviral Particles

The third-generation lentiviral packaging system was used for the generation of lentiviral particles, as previously described [21]. HEK293T cells were transfected with the lentiviral vector of choice and three different packaging plasmids (pMDLgpRRE, pRSVREV and pMD2VSVG) using FuGENE^®^ HD transfection reagent (Promega, Madison, WI, USA). Supernatants containing the viral particles were collected after 48, 72 and 96 h post transfection and passed through a 0.45-micron filter before being concentrated using polyethylene glycol and sodium chloride (NaCl). Viral particles were stored at −80 °C. The CCL2 knockdown was achieved by cloning shRNA into the pLKO.1 TRC vector (Addgene plasmid, Addgene, Cambridge, MA, USA) [66]. GBM cell lines (GBM1, JHH520, SF188) were transduced with lentiviral particles containing shβ-catenin/shCCL2 plasmids. Transfected cells were selected using 2 µg/mL puromycin (Sigma-Aldrich). The proliferation and migration assay were performed after stable conditions, and sufficient cell numbers were achieved between eleven to thirteen days after transduction. 

### 2.3. Cell Viability and Cell Death Assays

GBM cell lines were seeded in triplicates on 96-well-plates at a density of 1.5 × 10^4^ cells/mL and cultivated in 100 µL neurosphere medium for a total of six days. The viability was assessed using the Thiazolyl Blue Tetrazolium Bromide assay (MTT, Sigma-Aldrich), according to the manufacturer’s instructions. Absorbance was measured at 570 nm (reference 650 nm) using a Paradigm™ multiplate reader (Beckman Coulter, Brea, CA, USA). Cells were treated with MSAB at 1, 1.5 and 2.25 µM diluted in neurosphere medium for 24 h. Control cells were treated with vehicle (DMSO) only. To assess cell death after MSAB treatment, the MUSE Annexin V & Dead Cell Kit (Merck Millipore, Burlington, MA, USA) was used and cells were prepared according to manufacturer’s instructions. The analysis was performed using the MUSE cell analyzer (Merck Millipore). 

### 2.4. Clonogenicity Assay

To assess the clonogenic capacity of cell lines, we performed colony formation assay in soft agarose, as described previously [22]. Briefly, six-well plates were coated with a bottom layer consisting of 1.5 mL of 1% agarose (Life Technologies, Carlsbad, CA, USA) and neurosphere medium. A 2 mL layer consisting of 0.6% agarose containing 5 × 10^3^ cells/well was coated on top and it was covered with additional medium (2 mL). After 3 weeks of incubation under standard cell culture conditions, 1 mg/mL 4-Nitro tetrazolium chloride (NBT) solution (Sigma-Aldrich) was added to stain the colonies overnight at 37 °C. The experiments were quantified using Clono Counter software [67]. 

### 2.5. Quantitative Real Time PCR (RT qPCR)

RNA extraction was performed using the RNeasy Mini Kit (Qiagen, Hilden, Germany) according to the manufacturer’s instructions. RNA concentrations were measured photometrically using the Nanodrop2000 spectrometer (Thermo Scientific, Waltham, MA, USA). Two micrograms of RNA were utilized to synthesize complementary cDNA single strands using M-MLV reverse transcriptase (Promega, Madison, WI, USA) and random hexameric primers. Quantitative real time PCR was performed using advanced SYBR Green Supermix (BioRad, Hercules, CA, USA), 10 ng of cDNA and 10 pmol of each primer. Data were analyzed in a CFX Connect Thermocycler (BioRad). Relative expression levels of genes were normalized to the endogenous housekeeping gene β-actin. The Primer sequences can be found in Supplementary Table S1.

### 2.6. Whole Genome Transcriptome Analysis

Whole genome transcriptome analysis (3′mRNA sequencing) was performed at the NGS Core Facility (Bonn, Germany). The R package Deseq2 was applied to identify differentially expressed genes in the control cells versus the *β*-catenin knockdown cells. The R package clusterProfiler was used to view these differentially expressed genes enriched in the KEGG pathways.

### 2.7. Western Blotting

Cells were lysed in ice-cold RIPA buffer and protein concentrations were determined using the DC Protein Assay Kit (BioRad) following manufacturer’s instructions. Incubation with primary antibodies against active β-catenin (1:1000, BD Sciences, Franklin Lakes, NJ, USA) and β-actin or GAPDH (1:5000, Thermo Fisher) was performed overnight at 4 °C on a 3D-shaker in 5% BSA (VWR Life Science, Radnor, PA, USA) in TBST. As secondary antibodies, we used goat-anti-rabbit antibody IRDye800CW (1:10,000, LI-COR #926-32211, Lincoln, NE, USA) and goat-anti-mouse antibody IRDye680RD (1:10,000, LI-COR #926-68070) diluted in blocking solution and incubated for 1 h at room temperature. Signal detection was performed on a luminescence-based system in a LI-COR Odyssey CLx Imager (LI-COR). Luminescence values for active β-catenin were normalized to the corresponding GAPDH or β-actin values. 

### 2.8. ELISA

Cells were seeded at a density of 5 × 10^5^ cells/mL in neurosphere medium. Supernatants were collected after 24 h and passed through a 0.2 µM micron filter before being stored at −20 °C until needed. ELISA was performed using Human MCP-1 (CCL2) Standard ABTS ELISA Development Kit (Peprotech) following manufacturer’s instructions. ABTS Liquid Substrate (Sigma-Aldrich) was utilized and color development was measured at 405 nm with wavelength correction set at 650 nm using Paradigm™ multiplate reader (Beckman Coulter, Brea, CA, USA). Measured values were compared to obtain standard curves and normalized to total protein concentrations determined by DC Protein Assay Kit (BioRad). 

### 2.9. PBMC Migration Assay

The migration assay was performed using 6.5 mm diameter Transwell cell culture inserts (5 µm pore size; Costar, Washington, DC, USA; REF3421). Human PBMCs isolated from the blood of healthy donors were isolated by Ficoll density centrifugation, washed, counted and re-suspended in serum-free RPMI medium in the upper chamber of the filter (1 × 10^6^ cells in 500 µL). In the lower chamber, 800 µL of tumor-conditioned media was added. Cells were left to migrate for 4 h at 37 °C. Afterwards, cells remaining on the upper surface of the filter were removed with a cotton swab. Cells that migrated to the lower chamber were collected. Live cells were re-suspended in trypan blue and counted using a hemocytometer. 

In an additional experiment, PBMCs were harvested and stained with fluorescently labeled antibodies to assess monocyte migration. For this, PBMCs were stained with Zombie-Yellow Live/Dead stain, incubated with CD16/CD32 Fc blocking antibody and stained with an antibody against CD14 (FITC, 1:100 dilution). Samples were run on a Beckman Coulter Gallios flow cytometer and analyzed using the Kaluza 2.1 software. 

### 2.10. Luciferase Reporter Assay

To detect canonical Wnt pathway activity, we stably transfected GBM cells with a reporter construct containing seven TCF-binding sites followed by a firefly luciferase cassette as described previously [22]. Transfected cells were selected using 2µg/mL puromycin (Sigma-Aldrich). For each measurement, cells were harvested and washed in PBS. Cells were treated with MSAB at 10 µM diluted in neurosphere medium for 24 h. Control cells were treated with vehicle (DMSO) only. Cells were prepared according to manufacturer’s protocol (ThermoFisher Scientific, Madison, WI, USA). Luminescence readout was performed at 490 nm emission wavelength on Paradigm™ multiplate reader (Beckman Coulter, Brea, CA, USA) and normalized to ß-galactosidase activity.

### 2.11. Statistical Analyses

All data were obtained from three independent replicates and are shown as mean ± SD. Statistical significance was calculated using an unpaired student’s *t* test using *GraphPad Prism software*, version 8.0 (GraphPad Software, San Diego, CA, USA). Differences were considered significant for a *p* value of *p* < 0.05. 

## 3. Results

### 3.1. β-Catenin Expression by GBM Cells Impacts Monocyte Migration and CCL2 Secretion

To identify the impact of β-catenin in glioma cells on immune cell migration, we first established a β-catenin knockdown using small hairpin RNA (shRNA) interference in three GBM cell lines (GBM1, JHH520 and SF188).

**Figure 1:**
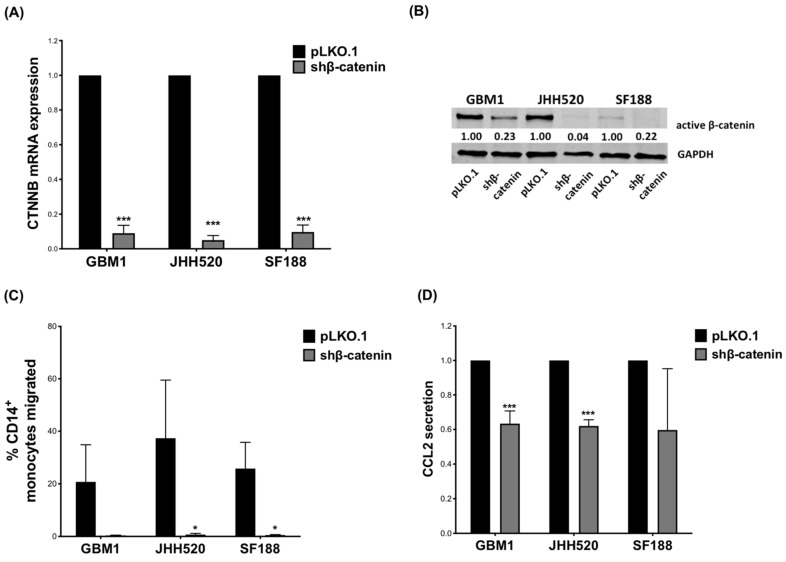
β-catenin knockdown in GBM cells reduces migration of CD14^+^ monocytes in vitro: (**A**) GBM cell lines (GBM1, JHH520, SF188) were transduced with lentiviral particles containing shβ-catenin plasmids and knockdown efficiency (relative mRNA expression) was confirmed using RT-qPCR and (**B**) Western blotting. (**C**) CD14^+^ monocyte migration towards TCM of β-catenin knockdown cells was decreased compared to migration towards TCM of control (pLKO.1) cells. **(D)** CCL2 levels in TCM of shβ-catenin GBM cells were measured after 24 h incubation by ELISA and compared to control cells (pLKO.1). The relative CCL2 secretion data are presented as mean ± SD (n = 3). Statistical significance was calculated with unpaired *t*-test. * *p* ≤ 0.05 *** *p* ≤ 0.001.

Reduced expression of *CTNNB1* (gene encoding β-catenin) was confirmed by qPCR (Figure 1A) and at protein level by Western blot (Figure 1B). Reduced β-catenin expression decreased proliferation of both GBM1 (Day 4: *p* = 0.049; Day 6: *p* = 0.015) and JHH520 cells (Day 2: *p* = 0.007; Day 4: *p* = 0.016) (Supplementary Figure S1A). We performed comprehensive gene expression analyses to determine the impact of β-catenin knockdown on GBM cell lines (Supplementary Figure S2). The analysis revealed several genes with altered expression (GBM1: n = 39 upregulated, n = 87 downregulated; JHH: n = 65 upregulated, n = 178 downregulated; SF188: n = 79 upregulated, n = 89 downregulated) (Supplementary Figure S2A,B). The results of KEGG pathway analysis showed that the differentially expressed genes (DEGs) were highly associated with signaling pathways ranging from N-glycan biosynthesis to metabolism and carcinogenesis in these cell lines (Supplementary Figure S2C). As expected, the expression of a large number of genes closely associated with the Wnt/β-catenin pathway was affected by knockdown of β-catenin (Supplementary Figure S3).

To investigate the effect of β-catenin in GBM cells on immune cell migration, we performed a Boyden chamber assay in which PBMCs migrated through a porous membrane towards media conditioned by GBM cells. Tumor conditioned media (TCM) derived from β-catenin knockdown GBM cells significantly reduced the number of migrated PBMCs compared to TCM derived from control cells (Supplementary Figure S1C). We next investigated the effect of β-catenin expression on monocyte migration. Similarly, TCMs collected from GBM cells with reduced β-catenin expression decreased monocyte migration significantly in JHH520 (*p* = 0.046) and SF188 (*p* = 0.012), whereas the reduction observed for shβ-catenin GBM1 cells did not reach statistical significance (Figure 1C). 

CCL2 is a strong chemoattractant for monocytes and has already been associated with β-catenin expression [47,68,69,70,71,72,73,74,75]. We therefore investigated how β-catenin expression affects CCL2 production. β-catenin suppression significantly decreased CCL2 gene expression in GBM1 (*p* = 0.0119) and JHH520 (*p* = 0.0432) cells (Supplementary Figure S1B) as well as CCL2 protein levels in TCM of GBM1 (*p* ≤ 0.001) and JHH520 cells (*p* = 0.0001) (Figure 1D). SF188 showed a similar, yet not statistically significant reduction in CCL2 protein, but not in the mRNA level. 

### 3.2. Recombinant CCL2 Restored PBMC Migration in shβ-Catenin TCM and CCL2 Knockdown Reduced Monocyte Migration

**Figure 2:**
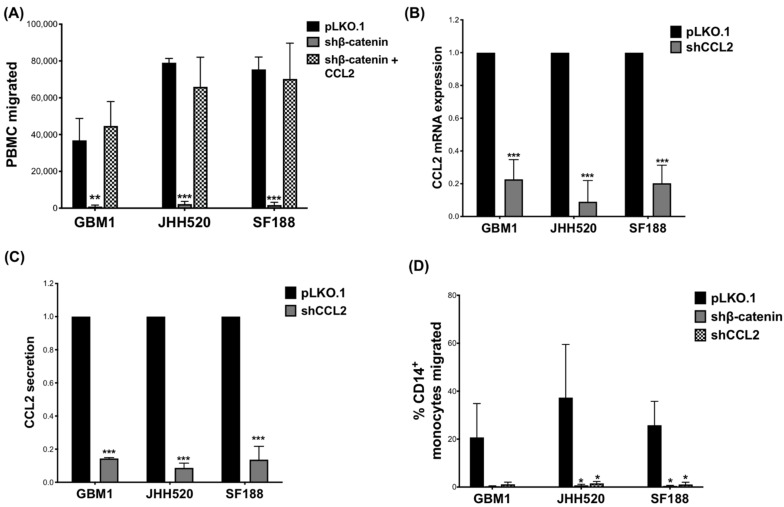
Recombinant CCL2 restored PBMC migration in shβ-catenin TCM and CCL2 knockdown reduced monocyte migration: (**A**) Recombinant CCL2 (100 ng/mL) was added to the TCM of β-catenin knockdown cells and restored PBMC-attracting ability. GBM cell lines were transduced with lentiviral particles containing shCCL2 plasmids and knockdown efficiency (relative mRNA expression and relative CCL2 secretion) was confirmed using (**B**) RT-qPCR and (**C**) ELISA, respectively. (**D**) CD14^+^-monocyte migration was decreased after treatment with TCM of shCCL2 knockdown cells compared to treatment with TCM of control (pLKO.1) cells. Data are presented as mean ± SD (n = 3). Statistical significance was calculated with unpaired *t*-test. * *p* ≤ 0.05, ** *p* ≤ 0.01, *** *p* ≤ 0.001.

To confirm that the observed decrease in CCL2 expression was responsible for the reduced migration of monocytes towards TCM from GBM cells with reduced β-catenin expression, we added recombinant CCL2 (100 ng/mL) to β-catenin knockdown TCMs, which restored PBMC migration (Figure 2A).

To investigate if a similar effect on monocyte migration can be observed in CCL2 knockdown GBM cells, we used shRNA to suppress CCL2 production. We confirmed reduced CCL2 gene expression (Figure 2B) and CCL2 secretion (Figure 2C) compared to control cells (pLKO.1). A slight decrease in proliferation was observed in CCL2 knockdown GBM cells, particularly of JHH520 cells (Day 2 *p* = 0.033, Day 4 *p* = 0.0171) (Supplementary Figure S4A–C). Indeed, similar to β-catenin knockdown, CCL2 knockdown significantly reduced CD14^+^-monocyte migration compared to control (Figure 2D, JHH520 (*p* = 0.049) and SF188 (*p* = 0.013). Again, in GBM1 the decrease did not reach statistical significance.

### 3.3. CCL2 Modulates Components of the Wnt/β-Catenin Pathway and Alters Clonogenicity of GBM Cells

**Figure 3:**
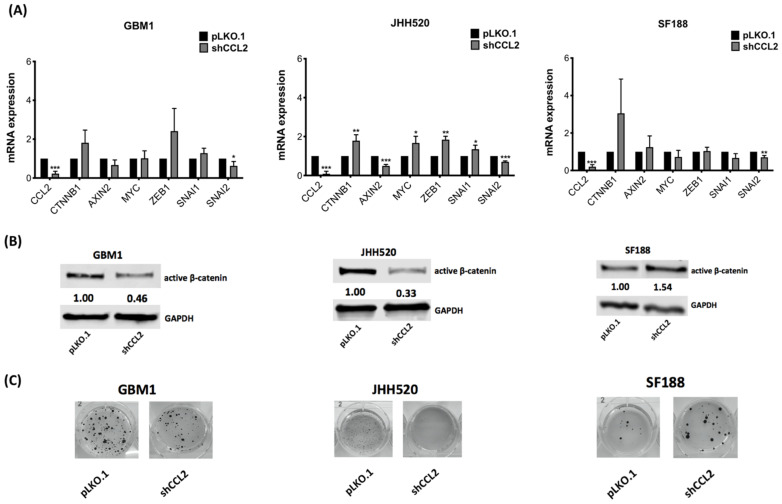
CCL2 knockdown alters expression of β-catenin target and related genes as well as clonogenicity of GBM cells: (**A**) β-catenin, the β-catenin target genes *Axin2*, *CCND1* and *c-Myc* and the β-catenin-associated genes *ZEB1*, *SNAI1* and *SNAI2* relative mRNA expression levels were analyzed by RT-qPCR in shCCL2 cells and compared to control cells (pLKO.1) (**B**) Non-phospho-(active)-β-catenin protein levels were detected using immunoblotting in shCCL2 and control cells (pLKO.1). (**C**) CCL2 suppression led to decreased clonogenicity of GBM1 and JHH520 while increasing clonogenicity of SF188 as detected by using a soft agar assay. Representative pictures of NBT stained colonies are shown. Abbreviations: NBT, 4-Nitro blue tetrazolium chloride. Data are presented as mean ± SD (n = 3). Statistical significance was calculated with unpaired *t*-test. * *p* ≤ 0.05, ** *p* ≤ 0.01, *** *p* ≤ 0.001.

To determine the effects of CCL2 on β-catenin activity, we further analyzed the phenotype of CCL2 suppressed GBM cells. We investigated the expression of β-catenin target (*AXIN2*, *MYC*) and further EMT–related genes (*ZEB1*, *SNAI1* and *SNAI2*). Following CCL2 knockdown, *CTNNB1* mRNA expression was upregulated, though not statistically significant in GBM1 and SF188. *SNAI2* expression was significantly reduced in all cell lines (Figure 3A). Gene expression of *AXIN2*, *MYC*, *ZEB1* and *SNAI1* was significantly different in JHH520 cells, but this could not be confirmed in GBM1 and SF188 cells (Figure 3A). Western blot analysis revealed significantly reduced β-catenin protein levels in GBM1 (*p* = 0.0013) and JHH520 (*p* = 0.0013) compared to the control cells (pLKO.1). In SF188 cells, CCL2 suppression significantly increased β-catenin protein levels (Figure 3B, *p* = 0.0003).

Next, we determined the clonogenic potential of CCL2 knockdown cells and found reduced colony-forming ability in GBM1 (not significant) and JHH520 (*p* = 0.0043), while the clonogenicity of SF188 was non-significantly elevated (Figure 3C and Figure S4D). To determine the effect of CCL2 on β-catenin activity, we treated GBM cells with recombinant CCL2 and analyzed the expression levels of *CTNNB1* and *AXIN2*. We observed elevated *CTNNB1* levels in GBM1 cells (*p* = 0.039) and increased *AXIN2* levels in JHH520 (*p* = 0.032). In SF188, *AXIN2* expression levels were downregulated (*p* = 0.0003) (Supplementary Figure S5A). Western blot analysis confirmed that active β-catenin protein levels were significantly increased after CCL2 treatment in GBM1 (*p* = 0.0064) and JHH520 cells (*p* < 0.0001) (Supplementary Figure S5B).

### 3.4. The β-Catenin Inhibitor MSAB Reduces Viability, Active β-Catenin Levels, Clonogenicity and Expression of β-Catenin Associated Genes in GBM Cells

To add a pharmacological model to our study, we tested the effects of the β-catenin inhibitor MSAB on GBM cells. MSAB has been shown to bind to β-catenin protein leading to its degradation [65].

**Figure 4:**
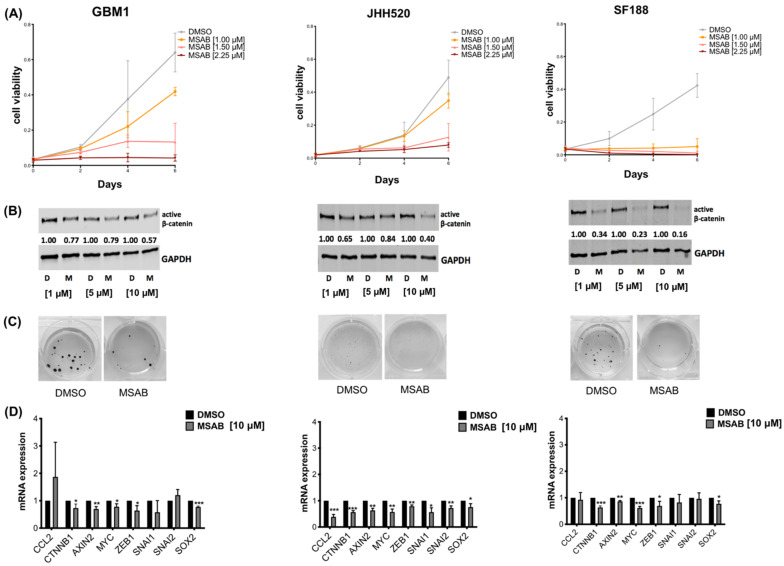
MSAB treatment reduces viability, active β-catenin protein levels and clonogenicity of GBM cells: (**A**) Cell viability was decreased by MSAB treatment in a dose-dependent manner. (**B**) Pharmacological β-catenin inhibition with MSAB led to suppression of non-phospho-(active) β-catenin in a dose-dependent manner as assessed by immunoblotting. Cells were treated with shown concentrations for 24 h. GAPDH was used as loading control. (**C**) MSAB treatment decreased clonogenicity of GBM cells in soft agar assay. Representative pictures of NBT stained colonies are shown. (**D**) The relative mRNA expression levels of β-catenin target genes (*Axin2*, *c-Myc*), -associated genes (*SNAI1*, *SNAI2*), neural stem cell marker *SOX2* and chemokine *CCL2* were measured by RT-qPCR in MSAB-treated cells compared to control cells (DMSO). Data are presented as mean ± SD (n = 3). Statistical significance was calculated with unpaired *t*-test. * *p* ≤ 0.05, ** *p* ≤ 0.01, *** *p* ≤ 0.001.

**Figure 5:**
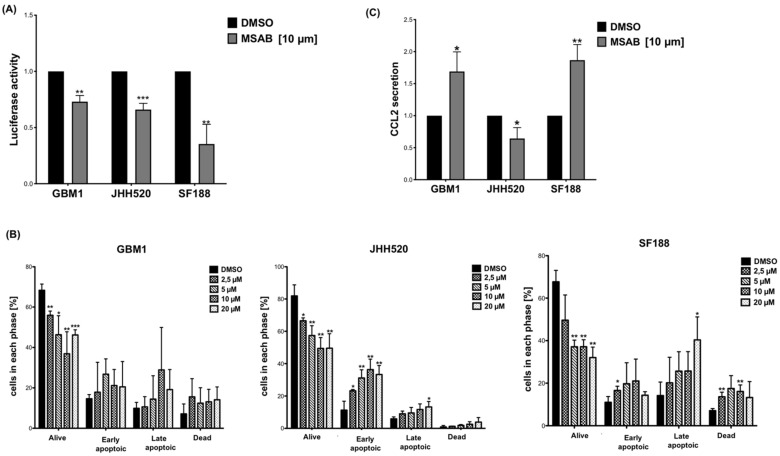
MSAB reduces Wnt-activity, induces apoptosis and modulates CCL2 secretion in GBM cells: (**A**) 24 h MSAB treatment (10 µM) reduced Wnt-activity in glioblastoma cell lines as assessed by Luciferase Reporter Assay. The relative luciferase activity data from three cell lines are shown. (**B**) 24 h treatment with MSAB induced apoptosis in GBM cell lines in a dose-dependent manner. Apoptosis was assessed with Muse Annexin V and Dead Cell Kit in three cell lines. (**C**) Altered CCL2 protein levels (relative CCL2 secretion) in the conditioned medium measured after 24 h incubation by ELISA. Data are presented as the mean ± SD (n = 3). Statistical significance was calculated with unpaired *t*-test. * *p* ≤ 0.05, ** *p* ≤ 0.01, *** *p* ≤ 0.001.

Thus, we treated GBM cell lines with MSAB and observed that it reduced cell viability in a dose-dependent manner (Figure 4A). Importantly, MSAB treatment also decreased active β-catenin protein levels in a dose-dependent manner (Figure 4B), confirming its β-catenin inhibitory effect. This was also evident in the soft agar clonogenicity assay, where a decrease in clonogenicity was observed after MSAB treatment (Figure 4C). Furthermore, treatment with MSAB for 24 h resulted in increased apoptosis of GBM cells (Figure 5B). To test whether the effect of MSAB was limited to β-catenin, we investigated the expression levels of its target genes using 10µM MSAB. Expression of *CTNNB1*, *AXIN2*, *MYC*, *ZEB1* and *SOX2* were significantly downregulated in all tested cell lines (Figure 4D). CCL2 expression was also downregulated in JHH520 cells (*p* = 0.0003), while we could not observe statistical significance in GBM1 and SF188. *SNAI1* and *SNAI2* were not altered by CCL2 knockdown in GBM1 and SF188.

### 3.5. MSAB Decreases Wnt/β-Catenin-Activity and Modulates CCL2 Secretion

We used a Luciferase Reporter assay driven by CTNNB1/β-catenin binding to multimerized TCF/LEF promoter sites, to measure canonical Wnt/β-catenin activity in the cell lines. Wnt-signaling was significantly reduced in all three cell lines after treatment with MSAB (Figure 5A). Furthermore, treatment with MSAB for 24 h resulted in increased apoptosis of GBM cells (Figure 5B). Similarly, treatment of GBM cell lines with MSAB altered secretion of CCL2 levels (Figure 5C). Treatment of GBM cells with MSAB (24 h) increased CCL2 levels in the supernatants of GBM1 (*p* = 0.0175) and SF188 (*p* = 0.0036), while the supernatant of JHH520 (*p* = 0.0224) showed significantly reduced CCL2 levels (Figure 5C). 

## 4. Discussion

In this study we showed that attraction of CD14^+^-monocytes by GBM is reduced by genetically targeting β-catenin in vitro. RNA interference of both β-catenin and CCL2 in GBM cells reduced migration of CD14^+^-monocytes towards TCM of glioblastoma cells. Furthermore, β-catenin knockdown decreased CCL2 secretion of glioblastoma cell lines, while CCL2 knockdown modulates β-catenin- and EMT-related genes. Pharmacological β-catenin inhibition with MSAB reduces Wnt/β-catenin activity and induces apoptosis in glioblastoma cells, while altering CCL2 secretion.

Therapy resistance and recurrence of GBM are associated with the presence of GSCs [5,6,7,8,9,10]. Previous studies have observed that β-catenin plays an important role in GBM, primarily by promoting growth, invasion, and treatment resistance by maintaining the stem cell properties [19,20,21,22,23,24,25,26,27,28,76,77]. Since β-catenin is involved in immunological processes [29,30,31,32,33], we investigated the effect of β-catenin on attracting immune cells, in particular CD14^+^-monocytes. GSCs were already associated with recruitment of tumor-supportive immune cells, such as TAMs and MDSCs, which derive from circulating monocytes [62,63,78,79]. TAMs in GBM have been shown to correlate with WHO grades [80] predicting the prognosis for high-grade glioma patients [81,82,83]. Interestingly, we observed that the treatment with TCM derived from β-catenin knockdown GBM cells reduced the migration of PBMCs and monocytes compared to control cells. Therefore, we hypothesize that β-catenin plays a key role in attracting precursor cells of TAMs/MDSCs to the tumor microenvironment.

In addition, we investigated whether CCL2 is involved in β-catenin-dependent cross talk between immune cells and GBM cells. Several studies emphasized the role of CCL2 in the GBM tumor microenvironment and in chemotaxis of tumor-supporting immune cells [47,48,49]. After β-catenin suppression we observed significantly reduced CCL2 levels in TCM of adult (GBM1 and JHH520) cell lines. The pediatric cell line SF188 showed a similar, but not significant effect. We added recombinant CCL2 to the TCM of shβ-catenin cells and observed that PBMC migration towards the TCM was restored. When comparing the effects of CCL2 and β-catenin knockdown on chemotaxis of monocytes, the two effects appear to be similar. These results suggest a pivotal involvement of CCL2 in β-catenin-stimulated PBMC attraction and in attraction of PBMC, in general. However, the exact mechanism of β-catenin-stimulated attraction of CD14^+^-monocytes remains to be investigated. In the pediatric cell line SF188 CCL2, protein secretion was not significantly decreased in β-catenin knock-down cells, while a decrease in monocyte migration was observed, indicating additional mechanisms involved in β-catenin-dependent monocyte attraction. In several studies, CCL2 was also associated with tumor cell migration and metastasis [84]. In line with this, we observed that CCL2 affects β-catenin- and EMT-related gene expression. Our finding regarding the interdependence of β-catenin and CCL2 is supported by a recent study showing that the β-catenin-CCL2 feedback loop mediates crosstalk between breast cancer stem cells and macrophages [64]. In addition, CCR2—the most common receptor for CCL2 [72,85]—promotes stabilization and translocation of β-catenin via AKT/GSK3β signaling in colon cancer cells [73]. Therefore, further studies are required to assess these mechanisms and whether the β-catenin/CCL2 axis can be found in other types of cancers.

To investigate whether pharmacological targeting of β-catenin would recapitulate our findings obtained with genetically modified GBM cells, we used MSAB, which binds β-catenin and induces its degradation [65]. Consistent with previous studies, MSAB treatment reduced the expression level of Wnt-related genes and Wnt-signaling activity of glioblastoma cell lines. We observed that MSAB treatment reduced GBM cell viability/clonogenicity of GBM cells and induced apoptosis in a dose-dependent manner, confirming the effectiveness of pharmacological Wnt/β-catenin-inhibition in our model [22]. Surprisingly, pharmacological inhibition showed different effects on CCL2 secretion than genetic modulation. We hypothesize this could be due to a difference in the duration or potency of β-catenin suppression. MSAB-induced β-catenin suppression was weaker than genetical suppression (Figure 1B and Figure 4B) and was performed over a shorter period. Further experiments with extended pharmacological suppression are required to verify these findings.

In this study we investigated the migration of CD14^+^-monocytes (precursors of TAMs and MDSCs) towards TCM. It remains to be determined how β-catenin and CCL2 affect differentiated TAMs, MDSCs and other immune cells of the GBM microenvironment. Therefore, further co-culture experiments with direct tumor-immune cell interactions and in vivo approaches are warranted to support our observations.

In our experiments, we observed differences between the three GBM cell lines. It is worth mentioning that differences in adult and pediatric cell lines can be expected due to the inherent heterogeneity of cancer cell lines, genetic/epigenetic variability, and/or inter-individual differences, as previously discussed [86]. For our comprehensive gene expression analysis, the effect of β-catenin knockdown on GBM cell lines showed clear differences. However, we found that multiple (though not completely overlapping) genes involved in the Wnt/β-catenin pathway were equally affected in all tested cell lines. 

Furthermore, we saw discrepancies between mRNA and protein data: for example, SF188 showed increased mRNA levels of CCL2 (Supplementary S1B), but decreased protein levels (Figure 1B) after β-catenin knockdown (both not significant). We speculate this could be due to posttranslational or epigenetic changes as well as possible protein-to-transcription feedback [87]. Therefore, more GBM cell lines should be tested to determine whether the transcriptional subtype of GBM cells (classical, proneural and mesenchymal) influences the response to β-catenin inhibition, and whether the efficacy of MSAB can be enhanced by additional CCL2 suppression. 

## 5. Conclusions

β-catenin and CCL2 are important determinants of monocyte attraction towards glioblastoma cells and show interdependence in vitro. Pharmacological β-catenin inhibition with MSAB decreases Wnt/β-catenin and leads to apoptosis in GBM cells.

## Data Availability

Not applicable.

## Supplementary Materials

The following supporting information can be downloaded at: https://www.mdpi.com/article/10.3390/ijms23094562/s1.
