# Supplementary material for: Crosstalk between β-Catenin and CCL2 Drives Migration of Monocytes towards Glioblastoma Cells
Source: Int J Mol Sci. 2022 Apr 20;23(9):4562. doi: 10.3390/ijms23094562 (PMC9101913; doi:10.3390/ijms23094562)
Supplement: Supplementary file 1 [file ijms-23-04562-s001.zip › ijms-1658045-supplementary.pdf]

International Journal of
*Molecular Sciences*

MDPI

**Supplementary figures:**

# Crosstalk between β-catenin and CCL2 drives migration of monocytes towards glioblastoma cells

**Philippe Aretz, Donata Maciaczyk, Suad Yusuf, Rüdiger V. Sorg, Daniel Hänggi, Hongjia Liu, Hongde Liu, Tikam Chand Dakal, Amit Sharma, Ramakrishna Bethanabatla, Silke Neumann, Jarek Maciaczyk**

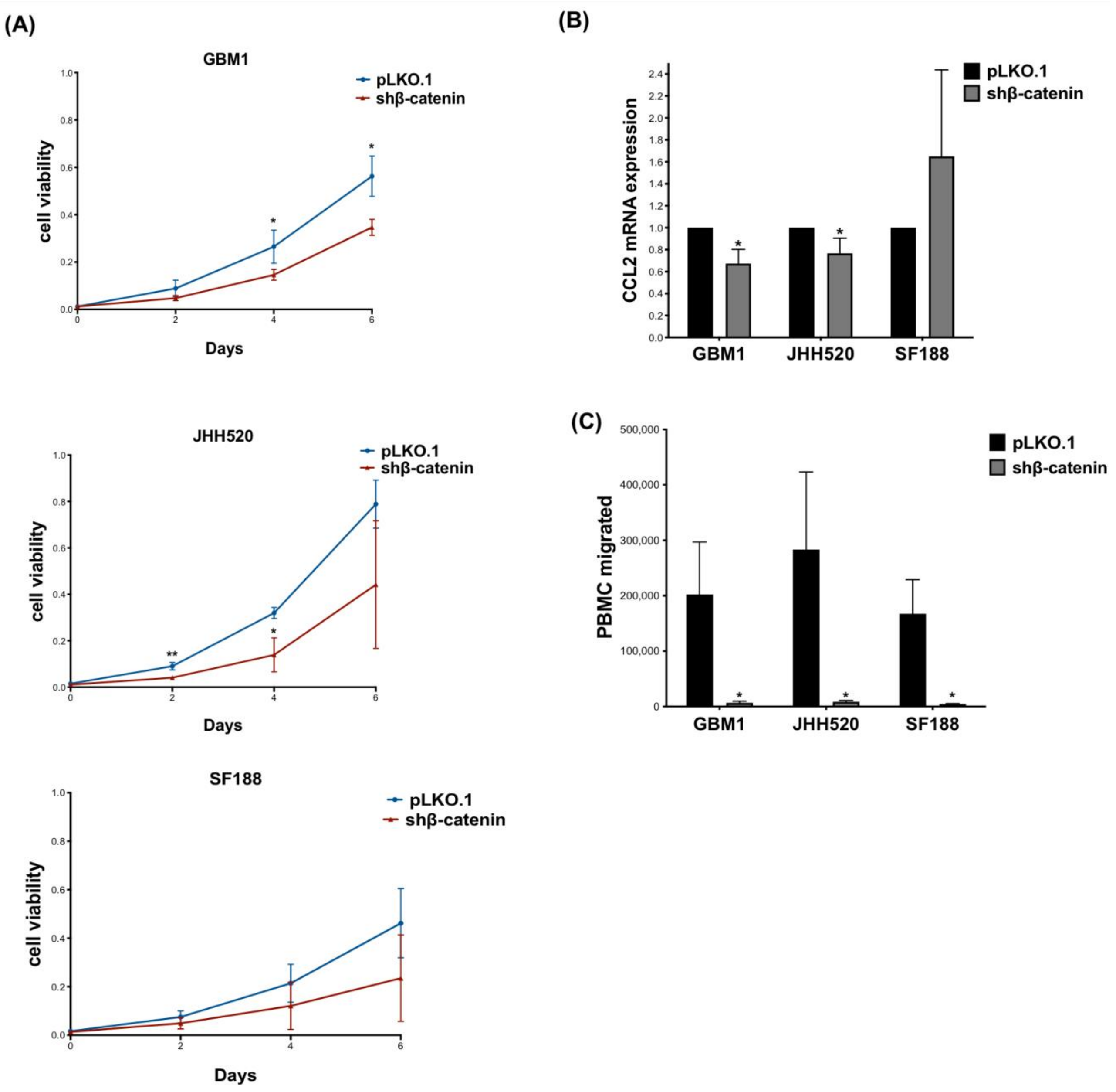


**Supplementary Figure S1: β-catenin knockdown partially decreases cell viability and CCL2 mRNA expression in GBM cells.** GBM cell lines (GBM1, JHH520, SF188) were transduced with lentiviral particles containing shβ-catenin plasmids: **(A)** Cell viability was partially reduced compared to control (pLKO.1) cells. Exponential growth curves were calculated for each condition. **(B)** The relative CCL2 mRNA expression levels were analysed by RT-qPCR in shβ-catenin cells and compared to controls (pLKO.1). **(C)** PBMC migration towards TCM of shβ-catenin GBM cells was significantly decreased compared to control (pLKO.1). Data are presented as mean ± SD (n=3). Statistical significance was calculated with unpaired t-test. * p ≤0.05 **p ≤0.01 ***p ≤0.001.

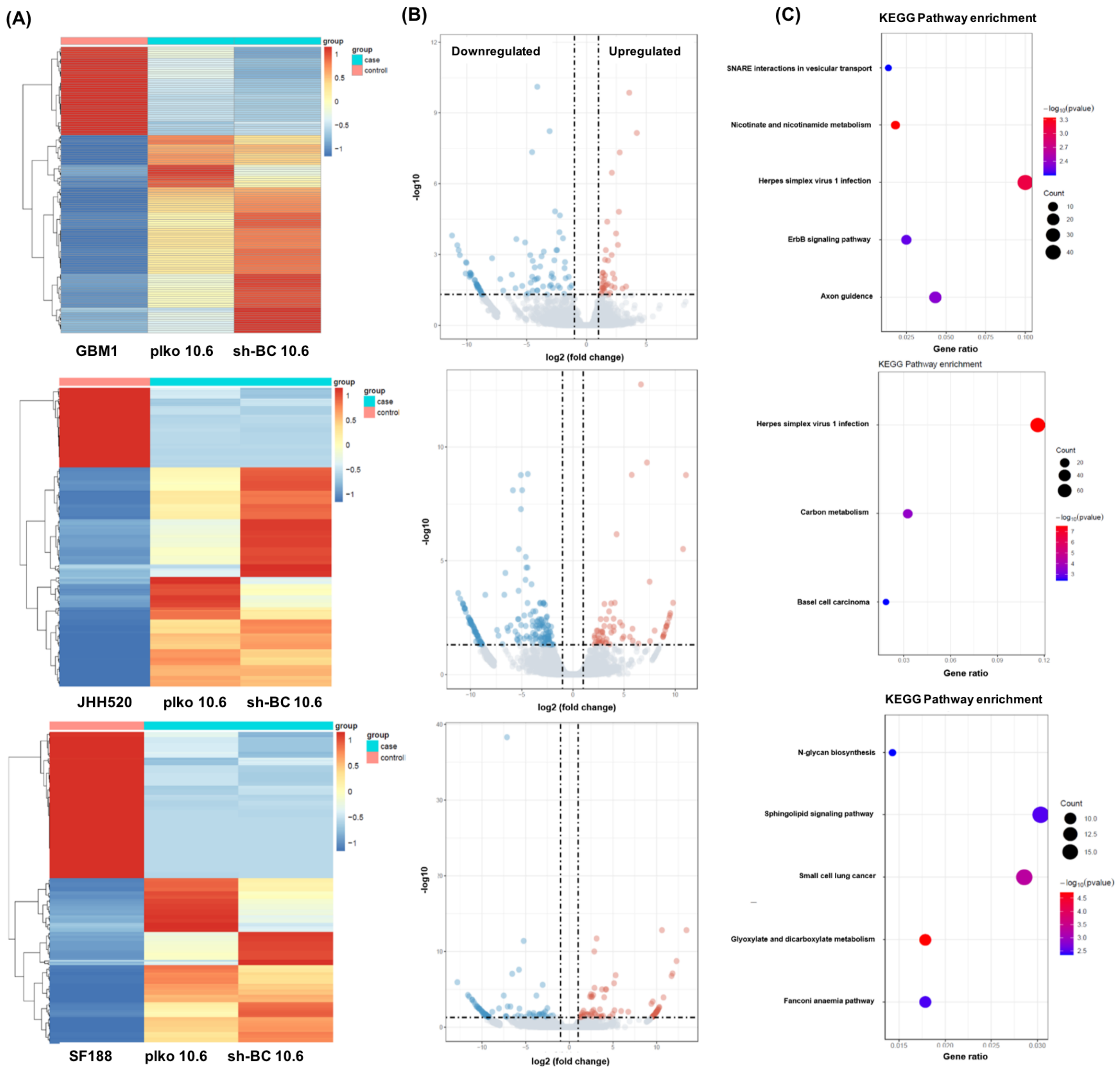


**Supplementary Figure S2:** Genome-wide gene expression analysis of three GBM cell lines: **(A)** heatmap showing variation of gene expression in control (pLKO.1) cells and shβ-catenin cells, **(B)** altered genes compared to the control cells (plKO.1), **(C)** KEGG associated pathways.

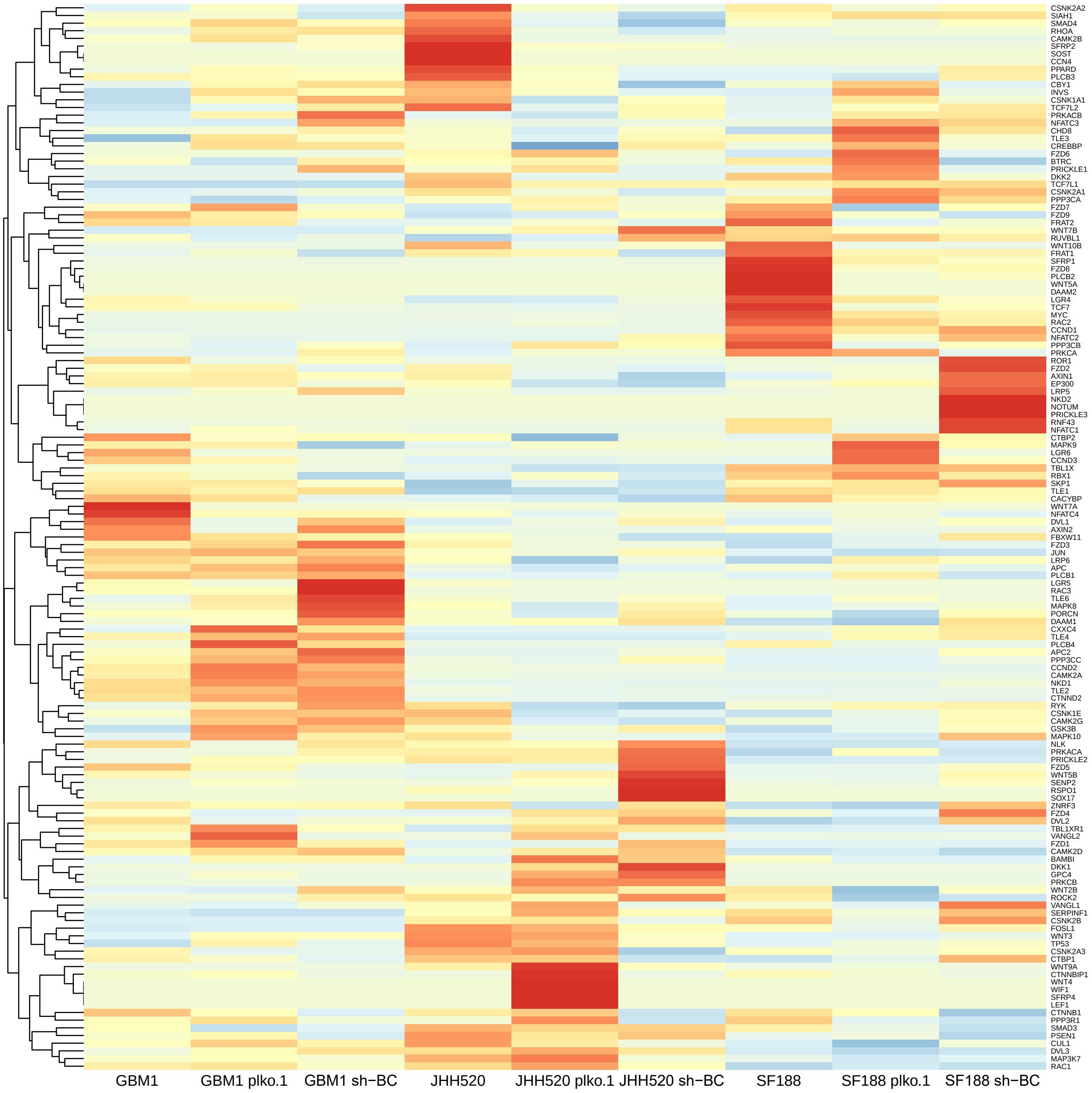


**Supplementary Figure S3:** A heat map shows changes in expression of genes involved in the Wnt/β-catenin pathway in three cell lines, control (pLKO.1) cells and shβ-catenin cells.

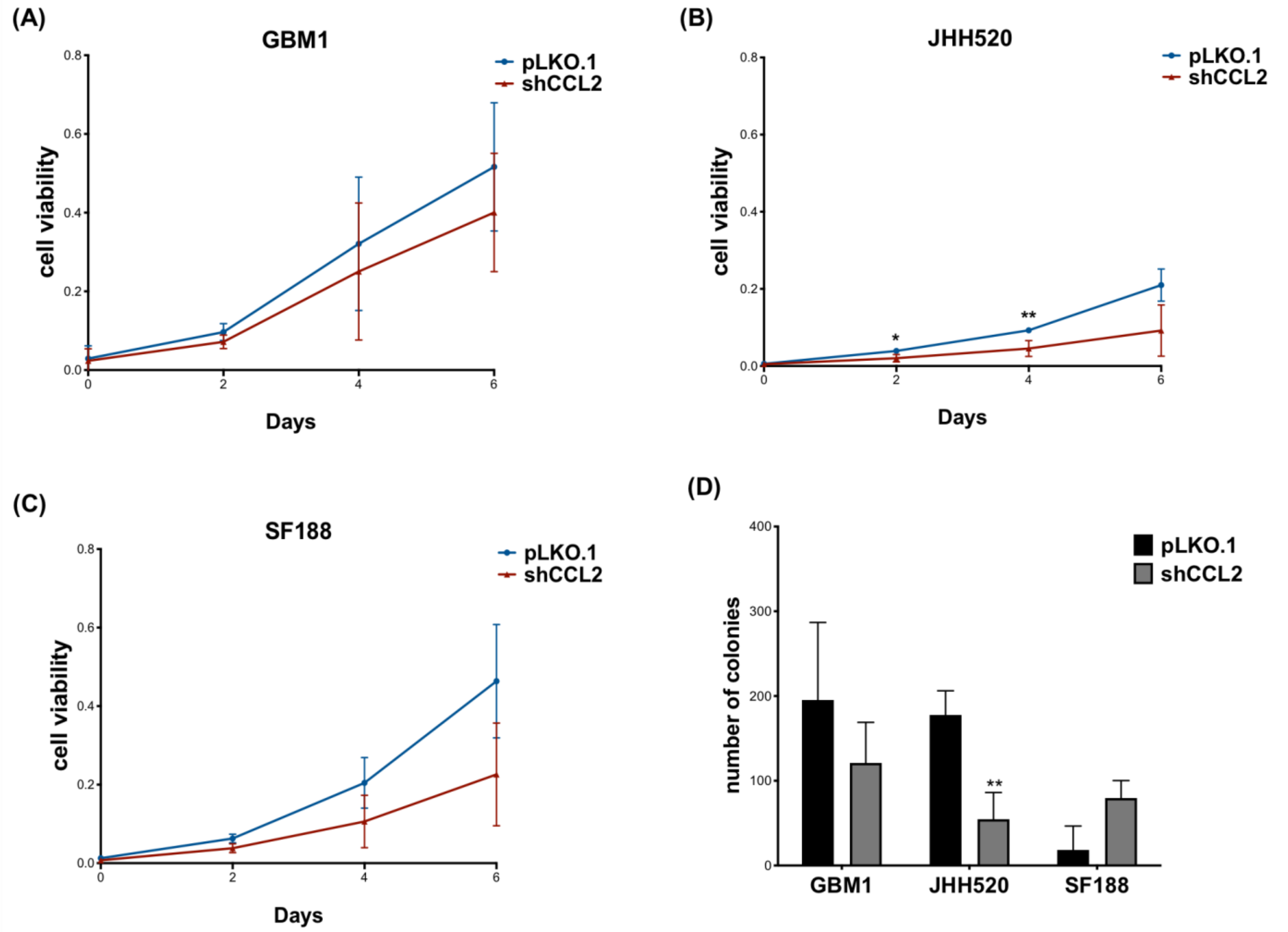


**Supplementary Figure S4: CCL2 mediated cell viability and clonogenicity of GBM cells:**
**(A)–(C)** Exponential growth curves representing cell viability in cell lines compared to control (pLKO.1) cells measured by MTT assay. **(D)** CCL2 suppression led to decreased clonogenicity of GBM1 and JHH520 while increasing clonogenicity of SF188 as detected by using a soft agar assay. Quantifications of three colony forming assays are shown. Data are presented as mean ± SD (n=3). Statistical significance was calculated with unpaired t-test. * $p \leq 0.05$ **$p \leq 0.01$ ***$p \leq 0.001$.

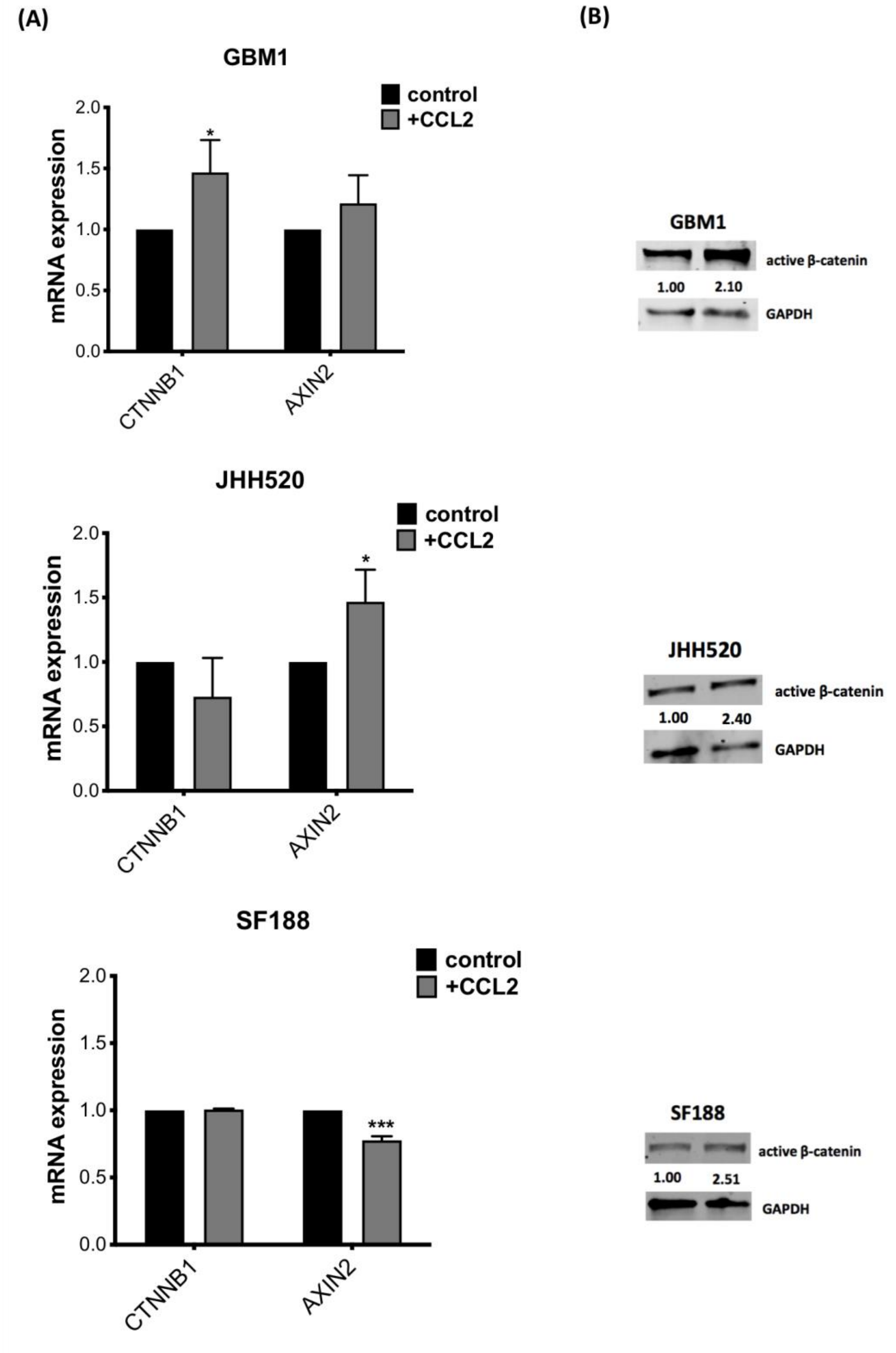


**Supplementary Figure S5: Treatment with recombinant CCL2 alters β-catenin -activity of GBM cells:** (**A**) The relative mRNA expression levels of β-catenin gene (*CTNNB1*) and target gene *AXIN2* were measured by RT-qPCR in CCL2-treated cells compared to control cells (PBS/BSA 0,1%). Cells were treated with 100 ng/ml recombinant CCL2 for 30 minutes. (**B**) Non-phospho- (active) β-catenin protein levels were increased after CCL2 treatment as assessed by immunoblotting. Data are presented as mean ± SD (n=3). Statistical significance was calculated with unpaired t-test. * $p \leq 0.05$ **$p \leq 0.01$ ***$p \leq 0.001$.

**Supplementary Table S1: Primer sequences used in RT qPCR**

| Primer | forward | reverse |
|---|---|---|
| **β-actin** | CCCAGCACAATGAAGATCAA | CGATCCACACGGAGTACTTG |
| **CTNNB1** | GGGCCTCAGAGAGCTGAGTA | TGAGCAGCATCAAACTGTGTAG |
| **AXIN2** | AGCCAAAGCGATCTACAAAAGG | GGTAGGCATTTTCCTCCATCAC |
| **c-MYC** | CCTTAATTAAAATGCCCCTCAACGTTAGCT | GGAATTCCATATGTTACGCACAAGAGTTCCGTA |
| **SNAI1** | GCTGCAGGACTCTAATCC | ATCTCCGGAGGTGGGATC |
| **SNAI2** | TGGTTGCTTCAAGGACACAT | GTTGCAGTGAGGGCAAGAA |
| **ZEB1** | AAGAATTCACAGTGGAG AGAAGCCA | CGTTTCTTGCAGTTTGGGCATT |
| **SOX2** | TGGACAGTTACGCGCACA | CGAGTAGGACATGCTGTA |
| **CCL2** | AGGTGACTGGGGCATTGAT | GCCTCCAGCATGAAAGTCTC |
